# Novel Insights of Effects of Pregabalin on Neural Mechanisms of Intracortical Disinhibition in Physiopathology of Fibromyalgia: An Explanatory, Randomized, Double-Blind Crossover Study

**DOI:** 10.3389/fnhum.2018.00406

**Published:** 2018-11-19

**Authors:** Alícia Deitos, Matheus Dorigatti Soldatelli, Jairo Alberto Dussán-Sarria, Andressa Souza, Iraci Lucena da Silva Torres, Felipe Fregni, Wolnei Caumo

**Affiliations:** ^1^Post-Gradaution in Medical Science at Medical School, Universidade Federal do Rio Grande do Sul, Porto Alegre, Brazil; ^2^Laboratory of Pain and Neuromodulation, Universidade Federal do Rio Grande do Sul, Porto Alegre, Brazil; ^3^La Salle University Center, Canoas, Brazil; ^4^Pharmacology Department, Instituto de Ciências Básicas da Saúde, Universidade Federal do Rio Grande do Sul, Porto Alegre, Brazil; ^5^Department of Neurology, Harvard Medical School, Boston, MA, United States; ^6^Berenson-Allen Center for Noninvasive Brain Stimulation, Department of Neurology, Beth Israel Deaconess Medical Center, Harvard Medical School, Boston, MA, United States; ^7^Anesthesiologist, Pain and Palliative Care Service, Hospital de Clínicas de Porto Alegre, Porto Alegre, Brazil; ^8^Surgery Department, School of Medicine, Universidade Federal do Rio Grande do Sul, Porto Alegre, Brazil

**Keywords:** fibromyalgia, cortical silent period, short intracortical inhibition, BDNF, S100B

## Abstract

**Background:** The fibromyalgia (FM) physiopathology involves an intracortical excitability/inhibition imbalance as measured by transcranial magnetic stimulation measures (TMS). TMS measures provide an index that can help to understand how the basal neuronal plasticity state (i.e., levels of the serum neurotrophins brain-derived neurotrophic factor (BDNF) and S100-B protein) could predict the effect of therapeutic approaches on the cortical circuitries. We used an experimental paradigm to evaluate if pregabalin could be more effective than a placebo, to improve the disinhibition in the cortical circuitries in FM patients, than in healthy subjects (HS). We compared the acute intragroup effect of pregabalin with the placebo in FM patients and healthy subjects (HS) on the current silent period (CSP) and short intracortical inhibition (SICI), which were the primary outcomes. Pain scores and the pain pressure threshold (PPT) were secondary outcomes.

**Methods:** This study included 27 women (17 FM and 10 HS), with ages ranging from 19 to 65 years. In a blinded, placebo-controlled clinical trial, participants were randomized to receive, in a cross-over manner, oral pregabalin of 150 mg or a placebo. The cortical excitability pain measures were assessed before and 90 min after receiving the medication.

**Results:** A generalized estimating equation (GEE) model revealed that in FM, pregabalin increased the CSP by 14.34% [confidence interval (CI) 95%; 4.02 to 21.63] and the placebo reduced the CSP by 1.58% (CI 95%; −57 to 25.9) (*P* = 0.00). Pregabalin reduced the SICI by 8.82% (CI 95%, −26 to 46.00) and the placebo increased it by 19.56% (CI 95%; 8.10 to 59.45; *P* = 0.02). Pregabalin also improved the pain measures. In the treatment group, the BDNF-adjusted index was positively correlated and the serum S100-B negatively correlated with the CSP, respectively. However, in the HS, pregabalin and the placebo did not induce a statistically significant effect in either intracortical excitability or pain measures.

**Conclusion:** These results suggest that pregabalin’s effect on cortical neural networks occurs, particularly under basal neuronal hyperexcitability, because its impact on the cortical excitability and the pain measures was observed only in the FM group. This indicates that pregabalin increased the CSP to induce inhibition in specific neural networks, while it increased the SICI to improve the excitability in other neurobiological systems. Trial registration in clinicaltrials.gov Identifier: NCT02639533.

## Introduction

Fibromyalgia (FM) is a syndrome that comprises of chronic widespread musculoskeletal pain, depressive symptoms, fatigue, sleep disturbance, and disturbances of the biological rhythm (Wolfe et al., 1990; Mease et al., 2005). Although the etiology of FM remains elusive, an appealing hypothesis is related to the overall hyperexcitement of the neurons based on central sensitization (CS) (Yunus, 2007; Latremoliere and Woolf, 2009; Woolf, 2011). The CS comprises a state that the central nervous system amplifies the sensory inputs in consequence to reinforcement on synaptic connectivity by structural and functional changes on axonic and dendritic terminals. The brain-derived neurotrophic factor (BDNF) plays a crucial role in these neuroplastic changes, and thus to the development and sustainment of CS pain. The FM is a CS syndrome in which patients have elevated serum levels of either S100-B protein or BDNF, which have been correlated with lower pain thresholds (Zanette et al., 2014). Additionally, in chronic pain, the increase in serum BDNF is associated with higher motor cortex disinhibition, indicated by a shorter cortical silent period (CSP) (Caumo et al., 2016).

In the clinical setting, the different individual symptoms and mechanisms of each CSP require a customized treatment approach. For this, we need to advance our understanding of alterations of neuroplasticity disease-related *in vivo*. A tool that provides insight to the neurotransmitter system, when we assess the cortical excitability, is transcranial magnetic stimulation (TMS) measures (Caipa et al., 2018). For example, FM patients, compared to healthy subjects (HS), showed a decrease in either short intracortical inhibition (SICI) or the CSP (Salerno et al., 2000). Since both SICI and CSP are mediated by inhibitory gamma-aminobutyric acid (GABAergic) interneurons within the primary motor cortex (Di Lazzaro et al., 2006; McDonnell et al., 2006), these findings have been interpreted as an indication for impaired GABA-mediated function in FM. Such metaplastic alterations were correlated with depression, catastrophizing, and fatigue scores in FM patients (Galhardoni et al., 2015).

As previously mentioned, the most clinical symptoms of FM have been linked to a defective inhibitory function. Pregabalin is a US Food and Drug Administration (FDA) approved drug for FM. According to *in vitro* studies, pregabalin exerts pharmacologic activity through direct interactions to a ligand of the alpha-2-delta subunit on voltage-gated calcium channels and reduces calcium influx at nerve terminals, resulting in a decreased release of several neurotransmitters, such as glutamate, norepinephrine, and substance P (Micheva et al., 2003; Staud and Spaeth, 2008). Although the TMS may be used to map cortical function and to measure the neuronal membrane excitability of human cerebral cortex, it does not permit to evaluate changes in cellular neurobiological processes directly (Ziemann et al., 1996a). In healthy subjects, pregabalin does not change the motor thresholds. This indicates that it does not predominantly effect changes of the neuronal membrane excitability (Ziemann et al., 1996b). At present, this disconnection between findings reported *in vitro*, in contrast with results found *in vivo*, is an overactivation of mechanisms that reduce excitation and lead to massive releases of GABA from inhibitory interneurons. Thus, the capacity of the GABA-uptake enzymes may be insufficient to remove GABA quickly from the synaptic cleft, thereby favoring postsynaptic GABAB-receptor activation (Di Lazzaro et al., 2004).

Therefore, it is plausible to consider the use of neuronal inhibition indexes to measure the effect of pregabalin on neural substrates, as this effect has been associated with a clinical impact on FM symptoms. Accordingly, the TMS paradigms permit that we evaluate differentially the inhibitory and excitatory neural arrangements, thus, we can measure how pregabalin affects cellular communication. In human studies, it is not possible to identify the direct effects of the drug on specific brain circuits, as it can only be identified on a remote neural network. Physiological measures such as TMS measures, therefore allow a more appropriate qualitative and quantitative evaluation of the effect of drugs in neurobiological systems, than plasma levels or dose rates do (Ziemann, 2004).

Although pregabalin is a drug widely used to treat FM, we have limited evidence that associates its effect in vivo, to the ratio of inhibitory/excitatory at the cortical level in FM. Therefore, to obtain new insights of the effect of pregabalin on the neural imbalance (excitability/inhibition) at the cortical networks in FM, we designed the present cross-over trial. We used an experimental paradigm to evaluate if pregabalin could be more effective than a placebo, to improve the neuronal imbalance (excitability/inhibition) in the cortical circuitries in FM patients in contrast to healthy subjects (HS). We compared the acute intragroup effect of pregabalin with the placebo, according to the condition: FM and HS in the current silent period (CSP) and SICI, which are the primary outcomes. The secondary outcomes are the pain pressure thresholds (PPT) and the temperature in °C that evokes a pain score of 6/10 on the numerical pain scale (0–10) during quantitative sensory testing (QST). We assessed if the basal neuronal plasticity state (i.e., levels of serum neurotrophins BDNF and S100-B protein) could predict the effect of therapeutic approaches on the cortical circuitries.

## Materials and Methods

### Study Design, Setting, and Participants

**FIGURE 1:**
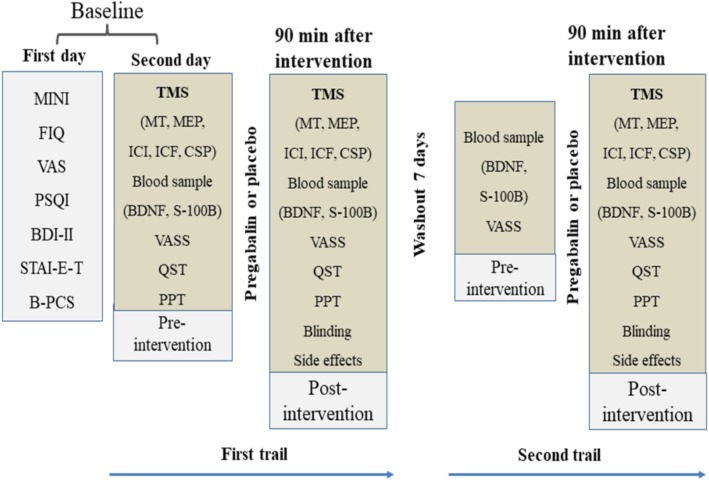
Experimental design – cross-over, assessments, and interventions in each one of two sessions. The period between each course was 1 week. Abbreviations: B-PCS, Brazilian Portuguese version of the pain catastrophizing scale; VAS, visual analog scale; STAI-E-T, state-trait anxiety inventory; BDI II, beck depression inventory II; FIQ, fibromyalgia impact questionnaire; PPT, pain pressure threshold; QST, quantitative sensory testing; MINI, mini-international neuropsychiatric interview; VASS, visual analog sleepiness scale; BDNF, brain-derived neurotrophic factor; S100B, S100 calcium-binding protein B; TMS, transcranial magnetic stimulation; TMS measures include motor threshold (MT), motor evoked potential (MEP), short intracortical inhibition (SICI), intracortical facilitation (ICF), and cortical silent period (CSP).

This randomized, double-blind, placebo-controlled crossover study was conducted according to the Declaration of Helsinki in Hospital de Clínicas de Porto Alegre (Rio Grande do Sul, Brazil). The protocol was approved by the IRB (IRB from the Hospital de Clínicas de Porto Alegre – HCPA/Approval number: 14-0624). All volunteers and patients provided written informed consent before participating in this study. Neither the patients nor the pain-free volunteers received monetary or any other compensation for participating in this study. The experimental design, cross-over, assessments, and interventions in each of the two sessions are presented in Figure 1.

### Participants

#### Pain-Free Control Volunteers

The volunteers were recruited from the general population by advertisement postings in universities, on the Internet, and in public places in the Porto Alegre area. Subjects were considered eligible to participate if they were female, right-handed, and between 19 and 60 years of age, and were screened for eligibility by phone. They answered a structured questionnaire that assessed the following variables: current acute or chronic pain conditions, use of analgesics in the past week, rheumatologic disease, clinically significant or unstable medical or psychiatric disorders, history of alcohol or substance abuse in the past 6 months, neuropsychiatric comorbidity, and use of psychotropic drugs. Volunteers responding positively to any of these questions and those with contraindications for TMS (Rossi et al., 2009) were excluded. Subjects with Beck depression inventory (BDI) (Warmenhoven et al., 2012) scores higher than 13 were also excluded (Beck et al., 1996).

#### Fibromyalgia (FM) Subjects

**FIGURE 2:**
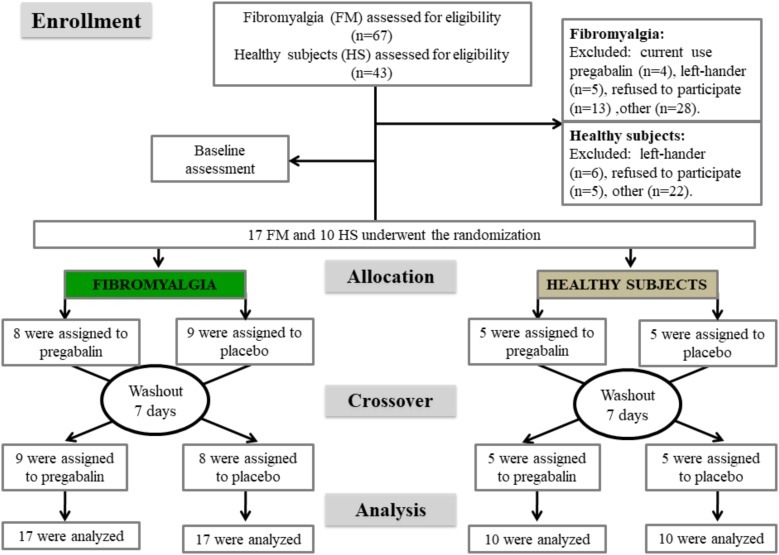
Flowchart showing recruitment and progress through the study.

FM subjects were recruited by directly contacting them from the institutional chronic pain clinic, by referrals from other clinic units, and through media advertising. FM diagnosis adhered to 2010 American College of Rheumatology criteria (Wolfe et al., 2011). All subjects were screened for eligibility by phone. We considered females who reported a pain score on the numerical pain scale (NPS 0–10) greater than 5, in the most days of the last month, were right-handed, and were aged between 19 and 60 years, eligible. The exclusion criteria were previous neurologic diseases, pregnancy, breastfeeding, illicit drugs use, history of alcohol abuse; current use of pregabalin, or failure to respond to pregabalin. We excluded subjects who did not understand Brazilian Portuguese and those with contraindications for TMS according to the guidelines for the use of transcranial magnetic stimulation in clinical practice and research (Rossi et al., 2009). The methods and results sections are reported according to the CONSORT guidelines items. Figure 2 shows the flowchart of the study.

### Sample Size

A superiority test from a crossover design, with a sample size of 16 subjects, divided into two groups with a 1:1: ratio, over two sessions, could test for the intragroup difference of 15% (*SD* = 8%) between the pregabalin or placebo group. The sample size was estimated for both primary outcomes (CPS and SICI) with a variation coefficient of 0.5, to test inequality and to achieve 90% power at a 0.1% significance. To account for the multiple outcomes and potential dropouts, we increased the sample size to 17.

### Interventions

The intervention involved one oral dose of pregabalin 150 mg acquired from ZODIAC as Prebictal^®^, in solid capsules containing pregabalin 150 mg and excipients. A placebo was manufactured with identical solid capsules containing starch. The capsules were manufactured in such a way that the placebo and active treatment had the same size, color, smell, and flavor. The TMS measures and pain measures were started 90 min after administering pregabalin, since an earlier study had demonstrated that a serum peak of pregabalin occurs at this time in patients when used orally (Bockbrader et al., 2010).

### Randomization

The randomization was generated by computer software by an investigator who was not involved with the assessments. Seventeen subjects with FM were allocated to receive pregabalin or a placebo in the ratio of 1:1. In this incomplete block or crossover trial, each subject received some interventions in two sessions. This means that the allocation in a cross-over manner, in the first and second sessions, was pregabalin (*n* = 8) and placebo (*n* = 9), respectively, and in the second session, pregabalin (*n* = 9) and placebo (*n* = 8), respectively. The allocation of the treatment of HS was performed in a similar manner. The experimental design and interventions in each session are presented in Figure 2.

### Blinding

To control for possible measurement bias, participants were instructed to discuss all aspects related to their treatment only with their treating physician (rather than the research personnel). Before the recruitment phase, opaque envelopes were sealed and numbered sequentially; they contained the treatment allocated. After the subjects agreed to participate in the trial, the envelopes were opened in sequence by a researcher who administered the medications. During the entire protocol timeline, an investigator not involved in the subjects’ evaluation was responsible for the blinding and randomization procedure. Other individuals who were involved in the participant’s assessments were unaware of the treatment group to which participants belonged. Further, to assess whether blinding was adequate, at the end of the experiment we asked participants to guess whether they had received pregabalin or a placebo and to rate their confidence in the answer on a Likert scale with five categories (“no confidence” to “completely confident”).

### Outcomes and Measurements

The primary outcome was the cortical excitability measured by the CSP and the SICI. The secondary outcome was the temperature in the QST that provoked a pain score 6 on the NPS (0–10) and the PPT.

#### Measurements of Cortical Excitability Using TMS

The motor cortex excitability was assessed using TMS with a MagPro X100 (MagVenture Company, Farum, Denmark) magnetic stimulator and a figure-8 coil. The coil was centered over the motor cortex (M1) and held tangentially to the scalp to reach the midline at 45°. To ensure the relaxation of arms and the correct positioning of the hand, subjects were asked to sit in a comfortable reclining chair. Cortical excitability parameters were registered through surface electromyography recordings gathered at the contralateral right first dorsal interosseous muscles using Ag/AgCl electrodes. First, the resting motor threshold (RMT) was assessed by obtaining five motor evoked potentials (MEPs) with a peak-to-peak amplitude of 50 μV out of 10 consecutive trials. After that, ten MEPs were recorded with an intensity of 130% of RMT. Moreover, the CSPs were assessed during muscle activity measured on a dynamometer set to approximately 20% of the maximal force. Accordingly, ten CSPs were recorded using an intensity of 130% of the RMT. SICI and intracortical facilitation (ICF) were measured using a paired-pulse TMS protocol, and the SICI was assessed using an interstimulus interval (ISI) of 2 and 4 ms; for the ICF measurement, the ISI used was 9 and 12 ms. For ICF and SICI, the conditioning stimulus (first) was set at 80% of the RMT while the test stimulus (second) was set at 100% of the individual MEP intensity, and the effect of the conditioning stimulus on the test stimulus was investigated (Kujirai et al., 1993). In total, 30 trials of paired-pulse were conducted in a randomized order (ten for each SICI, ICF, and control stimuli). We included the collection of all amplitudes of the MEPs, SICI, and SICF and the duration of the CSPs in an off-line analysis. SICI was taken as the mean percentage inhibition at ISIs of 2 and 4 ms, whereas SICF was taken as the mean facilitation at ISIs of 9 and 12 ms. The units for these parameters were: MEP in mV; SICI and ICF in their ratio to the MEP; and the CSP in milliseconds (ms) (Pascual-Leone et al., 1994). Two evaluators with specific training in performing TMS and all cortical excitability measurements preceding the pain assessment, conducted all measurements.

#### Pain Measurements

##### Pain from provocative test

Quantitative sensory testing was used to provoke a moderate pain [6/10 on the numerical pain scale (NPS)]. Moderate pain (6/10 NPS) was defined based on thermoalgesic stimuli delivered through a Peltier thermode of a surface of 30 × 30 mm^2^ (Schestatsky et al., 2011). The thermode was attached to the skin on the ventral aspect of the mid-forearm, and the temperature was increased at a rate of 1°C/s, from 30°C to a maximum of 52°C. The participants were instructed to press a button as soon as they felt moderate pain (6/10) on the NPS ranging from 0 (no pain) to 10 (the worst pain). The temperature necessary to evoke pain (score 6) of each patient was defined as the mean of three assessments performed with an interstimuli interval of 40 s (Schestatsky et al., 2011). A single training session was offered before the assessments to ensure that participants became familiar with the device. The thermode remained on the right ventral forearm, even though it was slightly altered on trials to avoid either response suppression or sensitization of the cutaneous heat nociceptors.

##### Pressure pain threshold (PPT)

The PPT was assessed using a digital algometer device (JTECH Medical Industries, Salt Lake City, UT, United States). The algometer’s 1 cm^2^ hard-rubber probe was pressed against the right antecubital fossa with a constantly increasing pressure. The procedure was stopped as soon as the subject indicated uncomfortable pain pressure (when the sensation of pressure changed to one of pain) and the PPT was recorded. This was repeated three times, and the average was calculated and used as the subject’s PPT.

#### Other Instruments and Assessments

The patients’ depressive symptoms were assessed using the Beck depression inventory II (Gomes-Oliveira et al., 2012). To evaluate the sleep quality of patients, the Pittsburgh sleep quality index was used (Bertolazi et al., 2011). The catastrophizing thinking related to pain was evaluated using the Brazilian Portuguese version of the pain catastrophizing scale (B-PCS) (Sehn et al., 2012). Anxiety was evaluated with the refined version of the state-trait anxiety inventory (STAI) (Kaipper et al., 2010). Psychiatric morbidity was defined according to the disorders included in the Mini-international neuropsychiatric interview (MINI) Brazilian version (Amorim, 2000). We used a standardized questionnaire to assess demographic data. An independent examiner was trained to administer the pain scales and to conduct the psychological tests. To evaluate the quality of life, we used the fibromyalgia impact questionnaire (FIQ) (Marques et al., 2006). The pain intensity was measured with a 100 mm visual analog scale (VAS). The VAS scores ranged from no pain (zero) to worst possible pain (100 mm). Participants were asked to answer the following question using the VAS of pain: (*i*) considering your pain, how intense was your worst pain during the last 24 h? Analgesic use of acetaminophen, non-steroidal anti-inflammatory drugs (NSAIDs), or opioid, was defined by the average of analgesics used during the previous week. For data analysis, analgesic use was included as a dichotomous variable (the use of analgesics on less than 4 days per week or use on more than 4 days per week). This approach was chosen because patients’ use of chronic pain-rescue analgesics changes each week, depending on their level of pain.

To measure the serum neuroplasticity mediators (BDNF and S100-B protein), we used standard procedures and collected blood at a minimum of 8 h after fasting early in the morning. All biological materials were collected before applying any intervention (pregabalin or placebo). Plastic tubes were centrifuged for 10 min at 5,000 *g* at 4°C. Serum was frozen at −80°C until assays were performed. BDNF and S100B serum concentrations were determined using specialized enzyme-linked immunosorbent assay (ELISA) kits (BDNF: catalog no. CYT306, the lower detection limit of the kit = 7.8 pg/mL, Chemicon/Millipore, Billerica, MA, United States; S100B: Millipore, MO, United States, catalog no. EZHS100B-33 K, the lower detection limit of the kit = 2.7 pg/mL).

### Statistical Analysis

To summarize the main characteristics of the sample, we used traditional descriptive statistics. To compare demographic and clinical measures between conditions (healthy or FM), we used a *t*-test for independent samples to compare continuous variables with parametric distribution and the chi-square or Fisher’s exact test for categorical variables. To test for normality, we used the Shapiro–Wilk test. To ensure that the data were normally distributed, we performed a log transformation for the BDNF level.

To examine the changes in the outcome measures across interventions (pregabalin and placebo), we applied a generalized estimating equation (GEE). The GEE analyses were conducted with an exchangeable working correlation structure to account for the correlation between the two sessions from a single participant (Ballinger, 2004). In the GEE model, the sequence of interventions (pregabalin or placebo) serves as the within-subject variable and accounts for the time variation among repeated measurements present in a longitudinal study design. The factors were the intervention types (pregabalin or placebo) and the conditions (FM patients or HS). In the final models, the interactions among the factors and sequence were also examined. For pairwise comparison of the predicted marginal means, multiple comparison tests were performed for each dependent variable separately. Cramér’s V was used as a measure of effect size for chi-square tests.

A MANCOVA model was used to assess the relationship between the BDNF and S100-B, according to the intervention group (pregabalin or placebo) on the SICI and CSP (dependent variables). Considering that pain severity, age, degree of depressive symptoms, analgesic use, and use of psychotropic medications are factors that can affect the biological process of BDNF secretion (Kuczewski et al., 2011; Molendijk et al., 2011), we constructed an adjusted index. A multivariate regression model controlled by multicollinearity was used to obtain an adjusted index used as the surrogate of the BDNF. Statistical significance was stated at a probability under 0.05 *P*-value. We used Bonferroni multiple comparison tests to adjust the differences for multiple comparisons. The data were analyzed using SPSS for Windows software version 22.0 (SPSS, Chicago, IL, United States).

## Results

### Patient Characteristics

Seventeen patients with FM and 10 HS were randomized in the study (Figure 2). The clinical and demographic features of patients are shown in Table 1. Regarding the side effects, in the pregabalin group, 85.18% (23/27) of the subjects presented minor side effects (MSE) (mild or moderate nausea, mild or moderate dizziness, mild or moderate dry mouth, mild or moderate headache, mild or moderate drowsiness, and mild xerostomia) and 11.11% (3/27) of subjects presented major side effects (MJSE) (severely blurred vision, severe dizziness, and crippling drowsiness). In the placebo group, 55.55% (15/27) of the patients presented MSE. The comparisons of the incidence of MSE between the pregabalin and the placebo groups were statistically significant (*P* < 0.035). However, neither the incidence of MSE nor the incidence of MJSE was significant when the groups (FM patients vs. HS) were compared (*P* > 0.05, for all comparisons). Blinding assessments were revealed by three subjects with FM and three HS; 22.2% (6/27) correctly guessed both interventions (pregabalin and placebo). Three reported to be “almost” and “completely” confident, one “moderately” confident, and two “somewhat” or “not confident at all” about the treatment that they had received.

**Table 1:** Sample characteristics.

Baseline characteristics	FM subjects *n* = 17	Controls *n* = 10	*P*-value
Age (years)	50.5 (8.7)	43.7 (9.4)	0.07
Body mass index (kg/m^2^)	31.3 (7.4)	24.2 (3.9)	0.00
Education (years)	10.1 (3.8)	17.3 (3.4)	0.00
Employed (yes/no)	10/7	10/0	0.26
Smoking (yes/no)	4/13	1/9	0.62
Pain on the VAS (24 h) (range 0–10)	7.1 (1.8)	NA	–
Pain on the VAS (last 7 days) (range 0–10)	7.9 (1.9)	NA	–
Pain duration (months)	152.47 (96.1)	NA	–
Antidepressants (yes/no)	14/3	NA	–
Anticonvulsant (yes/no)	3/14	NA	–
Benzodiazepine (yes/no)	4/13	NA	–
Analgesic doses (week)	28.2 (22.9)	NA	–
Beck Depression Inventory II (range 0–63)	25.4 (12.9)	3.8 (6.6)	0.00
Brazilian Portuguese Catastrophizing Scale (B-PCS) (range 0–52)	33.88 (12.0)	NA	–
State anxiety on STAI	27.3 (5.3)	17.9 (4.9)	0.00
Trait anxiety on STAI	29.35 (8.1)	16.7 (3.4)	0.00
Pittsburgh Sleep Quality Index (range 0–21)	12.6 (4.8)	4.2 (2.5)	0.00
FIQ (range 0–100)	70.39 (14.6)	NA	–
Physically active (yes/no)	10/7	6/4	0.95
Sedentary (yes/no)	7/10	4/6	0.95
**Psychiatric diagnosis using the Mini International Neuropsychiatric Interview (MINI)**			
Current depression (yes/no)	8/9	NA	–
Depression past (yes/no)	5/12	NA	–
Melancholic depression (yes/no)	7/10	NA	–
Bipolar I disorder (yes/no)	3/14	NA	–
Bipolar II disorder (yes/no)	2/15	NA	–
Generalized anxiety disorder (yes/no)	5/12	NA	–
Serum BDNF (ng/mL)	49.8 (16.3)	14.8 (6.9)	0.00
Serum S100-B (pg/mL)	18.99 (11.52)	25.99 (9.12)	0.00

*Data are presented as mean (SD), frequency, or proportion (n = 27). NA, not assessed. Abbreviations: B-PCP:S, Brazilian profile of chronic pain: screen; NPS, numerical pain scale; BDNF, brain-derived neurotrophic factor; VAS, visual analog scale; STAI, state-trait anxiety inventory; FIQ, fibromyalgia impact questionnaire. Of patients taking antidepressants, 52.9% used tricyclic; 47.1% selective serotonin reuptake inhibitor (SSRI), and 17.6% serotonin–norepinephrine reuptake inhibitors (SNRIs).*

### Primary Outcomes: Effects Concerning Cortical Excitability Measured by TMS (CSP and SICI)

As further analyses were conducted on paired data, means were assessed using the GEE approach and for pairwise comparisons. The between-group changes in cortical excitability measures (CSP and SICI) are shown in Table 2. The *post hoc* analysis indicated significant differences between the means between pregabalin and the placebo, in the CSP and SICI. However, in HS, these measures did not differ between pregabalin and the placebo (Table 2).

**Table 2:** Primary outcomes.

	Mean (*SD*)						
	Before intervention	After intervention	% (CI 95%)^¥^	Wald *χ*^2^	Df	*P*	Effect size
**Primary outcomes: cortical excitability measured**							
Cortical Salient Period (CSP)							
Fibromyalgia (*n* = 17)							
Placebo	65.79 (18.91)	64.75 (18.08)	−1.58% (−57 to 25.9)	7.48	1	0.00^∗^	0.68
Pregabalin	65.79 (18.91)	81.23 (15.37)	14% (4.02 to 21.63)				
Healthy (*n* = 10)							
Placebo	64.37 (31.35)	75.88 (34.52)	17.88% (2.22 to 21.93)	0.67	1	0.43	0.19
Pregabalin	64.37 (31.35)	77.02 (32.24)	16.42% (3.42 to 25.90)				
**Short Intracortical Inhibition (ratio: SICI/test stimulus)**							
Fibromyalgia (*n* = 17)							
Placebo	0.37 (0.20)	0.46 (0.18)	19.56% (8.10 to 59.45)	5.03	1	0.02^∗^	0.54
Pregabalin	0.37 (0.20)	0.34 (0.20)	−8.82% (−26 to 46.00)				
Healthy (*n* = 10)							
Placebo	0.29 (0.32)	0.33 (0.12)	12.12% (−24.00 to 41.00)	0.98	1	0.32	0.18
Pregabalin	0.29 (0.32)	0.31 (0.16)	6.45% (−23.00 to 38.00)				

*GEE analysis was performed to compare the effect of the interventions on CSP and SICI in fibromyalgia and healthy subjects. Data are presented as mean (SD) and mean difference after pre-intervention (n = 27). ^¥^Indicates the mean difference after pre-intervention. Df = degrees of freedom; ^∗^P < 0.05 indicates significant differences between treatment in the means adjusted for multiple comparisons by the Bonferroni test. Intracortical inhibition (ICI) expresses the relationship between the amplitude of wave and motor evoked potentials (relative amplitude, expressed in %), at interstimuli intervals (ISIs) of 4 ms with paired pulse. Cortical silent period (CSP) is expressed in milliseconds (ms). Pairwise comparisons of means were performed according to the concept of least-square means. Significant P-values are indicated in bold. CI, confidence interval. The effect size (ES) was computed in terms of Cramér’s V, also known as Cramér’s phi (ϕ) coefficients. The following guidelines were used to interpret the magnitude of the ES: small, 0.1 to 0.3; medium, 0.30 to 0.50; and large, 0.50 or higher. Values to be considered with clinical relevance should be a large ES.*

The baseline mean (SD) of the CSP in the FM and healthy groups was 65.79 (19.91) and 67.71 (12.42), respectively (*χ*2 = 0.63, DF = 1; *P* = 0.42). The mean (SD) in the SICI in FM and HS was 0.37 (0.20) and 0.29 (0.32), respectively (*χ*2 = 1.24, DF = 1; *P* = 0.26). GEE evaluation revealed that the order in which the intervention – pregabalin or placebo—was administered did not influence their effect either on the CSP (*χ*2 = 5.59, DF = 3; *P* = 0.13) or on the SICI (*χ*2 = 3.63, DF = 3; *P* = 0.30), that is, the first-order carryover effect did not occur.

The GEE model revealed that in FM from before to after intervention, pregabalin increased the CSP by 14%, while the placebo decreased it by 1.58%. It determined an effect size of large magnitude (0.68) (Table 2), with a potential clinical impact. In HS, pregabalin and the placebo produced an increase of 12.88 and 14.66% in the CSP, respectively. The effect was minimal, with no statistical difference or clinical relevance (Cramér’s V = 0.19).

**FIGURE 3:**
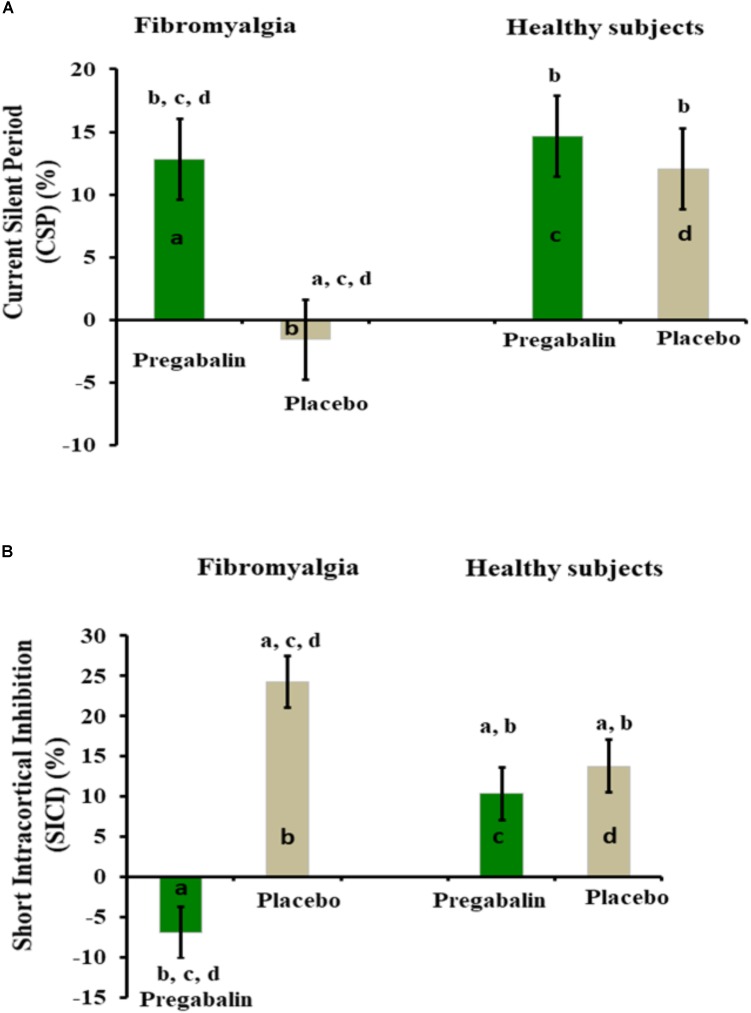
**(A,B)** Percentage of change from pre- to post-intervention. Bars indicate the mean and the standard error of the mean (SEM). The groups are identified by letters: FM group treated with pregabalin (a) and placebo (b). HS group treated with pregabalin (c) and placebo (d). All comparisons were performed by using a GEE model, followed by the Bonferroni correction for *post hoc* multiple comparisons. *Post hoc* differences between groups are indicated via superscript letters.

Pregabalin decreased SICI from the baseline by 8.10%, while the placebo increased it by 19.56%. Pregabalin produced a substantial size effect (0.54) (Table 2), with a possible relevance in the clinical setting. In HS, pregabalin and the placebo caused an increase of 10.34 and 13.79% in the SICI, respectively. The effect was minimal, with neither statistical difference nor clinical relevance. The change in means represented as percentages of CPS and SICI are shown in Figures 3A,B.

### Secondary Outcomes

#### Pain Measures: Provocative Test Performed to Provoke Pain Score 6 on the NPS (0–10) Using the Quantitative Sensory Testing (QST) and Pressure Threshold (PPT)

To compare the effect of interventions (pregabalin and placebo) on the means of the temperature that provoked pain score 6 on the NPS (0–10) by the QST as well on the PPT, we used the GEE approach (Table 3). GEE revealed that the order in which the intervention (pregabalin or placebo) was administered did not influence the effect of the intervention on the temperature that provoked pain to score 6 on the NPS (0–10) neither during the QST (*χ*2 = 1.71, DF = 3; *P* = 0.63) nor in the PPT (*χ*2 = 0.17, DF = 3; *P* = 0.68). That is, the first-order carryover effect did not occur. Although both the groups (FM patients and HS) were receiving pregabalin, it improves the threshold for the provocative test to produce pain score 6/10 on the NPS with a difference statistically significant only in the FM group.

**Table 3:** Secondary outcomes.

	Mean (*SD*)						
	Before intervention	After intervention	CI 95%	Wald χ^2^	Df	*P*	Effect size
**Secondary outcomes – treatment effect on pain outcomes**							
Secondary outcomes: pain measures							
Provocative test to induce pain score 6 on the NPS (0–10) using the QST (temperature °C)							
Fibromyalgia (*n* = 17)							
Placebo	42.22 (4.07)	40.27 (4.07)	(38.31 to 42.23)	18.0	1	0.00^∗^	1.05
Pregabalin	42.22 (4.07)	43.39 (4.36)	(41.10 to 45.28)				
Healthy (*n* = 10)							
Placebo	44.55 (5.52)	44.61 (3.58)	(42.89 to 46.33)	1.18	1	0.28	NE
Pregabalin	44.55 (5.52)	45.09 (3.62)	(43.35 to 46.83)				
Pain pressure threshold (kg/cm^2^)							
Fibromyalgia (*n* = 17)							
Placebo	2.44 (1.34)	2.30 (1.03)	(1.80 to 2.80)	5.46	1	0.01^∗^	0.57
Pregabalin	2.44 (1.34)	2.71 (0.90)	(2.12 to 3.07)				
Healthy (*n* = 10)							
Placebo	4.32 (1.03)	4.65 (2.63)	(3.38 to 5.92)	0.06	1	0.80	NE
Pregabalin	4.32 (1.03)	4.62 (2.39)	(3.47 to 5.78)				

*GEE analysis was performed to compare the effect of the interventions on pain measures in fibromyalgia and healthy subjects. Data are presented as mean (SD) (n = 27). Df, degrees of freedom; NE, non-estimated; ^∗^P < 0.05 indicates significant differences between treatment in the estimated marginal means adjusted for multiple comparisons by the Bonferroni test.*

Pregabalin increased the threshold for the provocative test to produce a pain score of 6/10 on the NPS from before to after intervention by 2.67% in the FM group. It was observed that the provocative test performed to produce a pain score of 6/10 on the NPS (0–10) resulted in an effect size with a large magnitude (Cramér’s V = 1.05) that is compatible with decreased pain perception (Table 3).

Pregabalin increased the PPT from before to after intervention by 9.96%. The size effect of pregabalin on the PPT compared to the placebo presents a large effect size (0.57) (Table 3), and in HS, the effect of pregabalin compared to that of the placebo produced a tiny size effect (0.08) (Table 3).

### Secondary Analysis: Relationship Between Serum Markers of Neuroplasticity Protein S100B and BDNF on the Effect of Treatment on Cortical Inhibition (CSP and SICI)

As presented in Table 1, serum BDNF and S100-B protein differ between healthy volunteers and FM patients. We observed that serum BDNF and S100-B in those with FM had significantly higher levels of both biomarkers in comparison to that in HS. To explore the effect of the intervention in cortical excitability to account for the neuroplasticity state before the intervention, we conducted an exploratory secondary analysis as presented below. First, considering the variables that comprise the confounding factor to be associated with either BDNF secretion and cortical excitability, we constructed an adjusted the index of BDNF, using a multivariate regression model to account for the influence of pain severity, age, depressive symptoms, analgesic use, and use of psychotropic medications. After the adjustment, the covariate retained in the model was the analgesic doses used weekly (r-squared = 0.24, standard β coefficient = −0.42, *t* = −3.36, *P* < 0.01). After constructing this BDNF-adjusted index, we ran a multivariate linear regression model with SICI and CSP as dependent variables, the BDNF-adjusted index, the serum S100-B protein, and their own interaction with treatment (pregabalin or placebo) as independent variables. The model is presented in Table 4. This analysis showed a significant relationship between the level of these markers and the CSP (Wilks’ λ = 0.37, *F* = 20.49, *P* < 0.001).

**Table 4:** Multiple regression models of the relationship between the effect of serum levels of S-100 protein and BDNF on the effect of interventions on the SICI and CSP (*n* = 17).

Dependent variable	Type III sum of squares	*df*	Mean square	*F*	*P*	Partial eta squared
Short intracortical inhibition (SICI)	0.46	4	0.12	1.77	0.17	0.22
Cortical silent period (CSP)	3881.54	4	970.38	4.77	0.005	0.43
	B	Std. error	t	*P*	CI 95%	
**Dependent variable: short intracortical inhibition (SICI)**						
Intercept	0.75	0.22	3.32	0.00	(0.29 to 1.21)	
Serum S100-B protein	0.01	0.022	0.63	0.53	(−0.03 to 0.06)	
BDNF adjusted index	−0.02	0.011	−1.78	0.09	(−0.04 to 0.003)	
Interaction						
Serum S100-B protein^∗^ treatment	−0.05	0.012	−0.36	0.71	(−0.06 to 0.02)	
BDNF adjusted index ^∗^ treatment	0.06	0.006	1.01	0.32	(−0.08 to 0.02)	
**Dependent variable: cortical silent period (CSP)**						
Intercept	62.12	12.61	4.93	0.00	(36.15 to 88.09)	
Serum S100-B protein	−2.68	1.24	−2.16	0.04	(−5.24 to −0.12)^∗∗^	
BDNF adjusted index	1.41	0.601	2.33	0.02	(0.16 to 2.65)^∗∗^	
Interaction						
Serum S100-B protein^∗^ treatment	0.84	0.69	1.22	0.23	(−0.57 to 2.25)	
BDNF adjusted index ^∗^ treatment	−0.43	0.31	−1.38	0.18	(−1.08 to 0.21)	

^∗∗^Interaction between dependent variable (S100B or BDNF)*trearment (factor).

The multiple regression analysis showed that the CSP, after the intervention, is correlated with serum levels of markers before the intervention. It was positively associated with the BDNF-adjusted index and negatively correlated with serum S100-B (Table 4). We did not observe an interaction between interventions (pregabalin or placebo) and the BDNF or S100-B (*P* > 0.05). This preliminary result suggests that BDNF and S100-B protein are correlated with the CSP, but these neurotrophic factors do not influence the effect of interventions (pregabalin or placebo) in SICI.

## Discussion

Our findings attempt to define the neural mechanisms at corticomotor pathways as a novel avenue to understanding the mechanisms involved in the central disinhibition in FM, as indicated by the effect of pregabalin on CSP and SICI. Additionally, in FM, the effects of pregabalin enhanced the PPT, and it improved pain perception. These results suggest that an interaction occurred between the disinhibition at motor cortex and the modulation of pain in FM and its behavioral correlates. In addition, they show that the baseline serum levels of neurotrophic factors as BDNF and S-100B protein might influence different responses on the corticomotor excitability as demonstrated by their correlations with the CSP.

The acute effect of pregabalin on cortical excitability parameters (i.e., CSP and SICI) and pain measures was distinct in FM patients compared to HS. In FM patients, the pronounced effect of pregabalin in the inhibitory cortical circuits as measured by CSP highlight its impact on motor cortical inhibition. However, in HS, neither the placebo nor pregabalin increased cortical inhibition (Figure 3A). These results support the concept that pregabalin is more effective in modulating nociceptive transmission when the neural excitability in the nociceptive input is enhanced. Similarly, the analgesic and anxiolytic effects of pregabalin occur particularly under conditions of hyperexcitability (i.e., pain and anxiety) to reduce the release of excitatory neurotransmitters and peptide neuromodulators (Tanga et al., 2006), leading to an overbalance of inhibition with proportional releases of GABA from the inhibitory interneurons.

The increase in the duration in CSP in FM patients involves the GABAB receptors (Siebner et al., 1998; Werhahn et al., 1999) and can reveal the extent of deactivated areas by neuronal hyperexcitability. This hypothesis is plausible according to studies with a cognitive task of increasing difficulty, as well as experimental pain studies that assess the change perception of pain when the resource attentional shift toward regions activated (Coghill et al., 1999). In addition, a previous study with FM patients showed that pregabalin reduced the signal evoked in functional magnetic resonance imaging in areas involved in pain processing (i.e., insula, thalamus, and precuneus) (Kim et al., 2013). In the same way, two other findings suggest the effect of pregabalin in pain processing: first, the absence of a similar modulation during the placebo use in FM patients, and second, the lack of pregabalin-induced changes on CSP in HS. Therefore, the effect of pregabalin on pain measures can be interpreted in the sense that, the disinhibition state involves multiple inhibitory mechanisms, which can change the sensory signals to the pain matrix. This result suggests that its effects enhance the balance of the excitatory/inhibitory systems in the corticomotor area.

The reduction in SICI induced by pregabalin indicates a mediated effect by GABAA receptors. It is important to note that this effect of pregabalin in SICI was observed only in the FM, and we did not find a significant effect of pregabalin on the cortical excitability in pain measures in HS. However, in a previous study, a similar result was found in HS with a dosage of 300 mg (Lang et al., 2006). The disagreement, related to pregabalin’s effect on HS, may be explained by the difference in the dosage. Our results may be of clinical relevance because they can help create a roadmap for customizing treatment in FM, based on an individual’s characteristics. The results are relevant to investigate novel therapeutic approaches that target the motor cortex (i.e., transcranial direct current stimulations tDCS and TMS), which appear to be a key system for the endogenous modulation of pain. Accordingly, the results suggest that M1 may be an entry port to assess the complex pain-related neural network, as well as to understand the role of M1 to inhibit or interrupt pain signals. Furthermore, they strengthen the notion that the use of neurophysiological measures in combination with neuropharmacological challenges provide an ideal opportunity to determine the dose, to produce a specific effect on the distinct brain systems involved in pain processing.

We observed that the placebo effect increased the SICI in FM, suggesting that expectancy raises corticospinal excitability. Accordingly, previous studies showed that the expectancy-induced increases in the MEP amplitude, that could result from the decreased excitability of intracortical inhibitory networks, or the increased excitability of intracortical facilitatory systems (van Elswijk et al., 2007). Another study on pain has shown that verbal instructions to participants, to focus on one body part, enhanced the placebo response (Geers et al., 2006). This data set suggests that different verbal information can direct attention toward or away from the body which can impact the response to the placebo. The assumption here is that the expectation induced through the placebo procedure can influence cognitive processing (Geers et al., 2006). However, we realize that we did not find this effect in the HS placebo group. We do not know any apparent reason to explain this finding; it is possible that a stimulus related to pain or treatment promotes selective attentional activation of neural networks and this justifies the higher cortical excitability observed in the FM placebo group.

As previously mentioned, the excitation of the primary motor cortex’s (M1) pyramidal neurons, is a target to a goal-setting process used to determine the intervention outcomes using a non-invasive method, such as magnetic pulses, to stimulate a restricted part of the cortex. In this context, the effect of pregabalin might help characterize the functional dysconnectivity of cortical networks that have been identified as important to assess specific aspects of the motor cortex function in pain physiopathology. At the same time, the effect of pregabalin in CSP and SICI is translated onto the intracortical circuitry within the motor cortex. This effect of pregabalin within M1 suggests that pregabalin modulates the corticospinal output. The thalamus is an important structure that mediates different components of pain and is also involved in the descending inhibition to modulate nociceptive inputs at the dorsal horn of the spinal cord. Thus, this sum of findings supports the hypothesis that the effect of pregabalin on pain and in cortical inhibition occurs by its modulatory force, in the thalamocortical connections projected to the primary somatosensory cortex. These results are in line with the notion that, inhibition is physiologically separate from excitation and has a lower threshold than that of excitation.

The correlation between CSP with BDNF and S-100B protein provide some evidence that these neuroplasticity factors might show at some level, the changes in the excitatory/inhibitory balance in the CNS involved in the transmission of nociceptive inputs. The BDNF is positively correlated to CSP; this suggests that the BDNF is involved in motor cortex disinhibition. This finding agrees with a recent study which found a positive correlation between SICI and the BDNF. It suggests that an interaction of this neurotrophic factor with the disinhibition of the motor cortex exists (Caumo et al., 2016). This result can reflect the BDNF influence on the GABAA receptors modulation (Ziemann et al., 2015). On the other hand, the correlation of the BDNF with the CSP also suggests its relationship to the GABAB receptor. According to experimental evidence, the GABAB receptor activation triggers the BDNF release and promotes the functional maturation of GABAergic synapses that increases the level of GABAA receptors at the plasma membrane (Kuczewski et al., 2011). The negative correlation of the S-100B protein with the CSP suggests that it is allied with the motor cortex excitability. Although the exact mechanism underlying this association is unclear, this is a plausible hypothesis because, the S-100B protein can increase intracellular free calcium concentrations to regulate neuron excitability (Selinfreund et al., 1991; Barger and Van Eldik, 1992). S-100B is the hallmark of astrocytic activation, and it might stimulate the astrocyte proliferation *in vitro* (Selinfreund et al., 1991; Barger and Van Eldik, 1992); in addition, experimental studies have demonstrated that GABAB receptors are expressed on cultured astrocytes (Selinfreund et al., 1991). Additionally, an experimental study on neuropathic pain showed that it is related to allodynia (Tanga et al., 2006). However, in clinical studies, we cannot isolate the effect of each system; for that reason, we cannot affirm that these are of the cause–consequence relationship type.

Several issues concerning the design of our study must be addressed. First, the absence of first-order carryover effects showed that the sequence in which participants received the intervention was not aliased with treatment differences. The crossover design permits to increase the uniformity, and it eliminates the between-subject variability because, the interventions under investigation were evaluated within the same subject (Grizzle, 1965). Given that participants act as their controls, the analyses could be based on paired data (using paired tests) (Brown, 1980; Maclure, 1991). Second, the sedative effect of pregabalin and its side effects can affect the blinding. The sleepiness might affect mainly the pain measures; however, the sleepiness score was included in the model and its influence did not statistically affect the outcomes related to pain. Third, the rate of guessing about the intervention (pregabalin or placebo) was similar between healthy and FM subjects. Additionally, our objective surrogates were less prone to bias, i.e., cortical excitability measures and pain provocation tests, and it was unlikely that un-blinding would change our conclusions. Fourth, we included only females because FM is more prevalent in women. Women are prone to activation upon negative emotional responses (i.e., stress, fear, and anxiety) and a higher anxiety trait has been associated with an imbalance in the excitability of the corticospinal tract (Vidor et al., 2014). Fifth, although a limitation of the current study was that we used the single baseline, and as psychophysical parameters are subject to a sizeable intersession variability, the post-treatment changes would be more comparable with the within-session baseline. Hence, this is a limitation that must be taken into account in the interpretation of our findings. However, we chose a cross-over design in which we had a single-subject design, to permit that the subjects serve as their control. This way, it is possible that this factor could have a lesser impact on our results. Finally, although the present findings are important to understand the possible neurobiological mechanisms of central excitatory/inhibitory balance systems in fibromyalgia, they do not support therapeutic decision-making in clinical settings.

These results suggest that pregabalin’s effect on cortical neural networks occurs, particularly under basal neuronal hyperexcitability, because its impact on the cortical excitability and in pain measures was observed only in the FM group. They suggest that pregabalin can reduce the inhibitory connections in specific neural networks, while it can increase the excitatory activity in others. However, they indicate that pregabalin effect is dependent on the baseline neuroplasticity state.

## Ethics Statement

This study was carried out in accordance with the principles of the Declaration of Helsinki and recommendations of the Ethics and Research Committee of Hospital de Clínicas de Porto Alegre, according to Brazilian Laws for Clinical Research in Humans. The protocol was approved by the Ethics and Research Committee of Hospital de Clínicas de Porto Alegre.

## Author Contributions

AD conceived the study, participated in its design and coordination, participated in the sequence alignment, and drafted the manuscript. MS participated in the design of the study, performed the statistical analysis, and drafted the manuscript. JD-S participated in the sequence alignment and drafted the manuscript. AS participated in the sequence alignment. IST participated in the design of the study and performed the statistical analysis. FF participated in the study design and coordination and helped to draft the manuscript. WC conceived the study, participated in its design, sequence alignment and coordination, and helped to draft the manuscript.

## Conflict of Interest Statement

The authors declare that the research was conducted in the absence of any commercial or financial relationships that could be construed as a potential conflict of interest.
